# Characterizing brain structures and covert vascular brain injury associated with burden and clusters of multimorbidity in older adults: A population‐based MRI study

**DOI:** 10.1002/alz70855_101849

**Published:** 2025-12-23

**Authors:** Yifei Ren, Xiaodong Han, Lin Song, Jiafeng Wang, Mingqing Zhao, Na Tian, Shi Tang, Min Zhu, Keke Liu, Yun Liu, Lidan Wang, Xinrui Zou, Jiahui Li, Xueran Ding, Cuicui Liu, Tingting Hou, Yongxiang Wang, Lin Cong, Yifeng Du, Chengxuan Qiu

**Affiliations:** ^1^ Department of Neurology, Shandong Provincial Hospital, Shandong University, Jinan, Shandong, China; ^2^ Shandong Provincial Clinical Research Center for Geriatric Neurological Diseases, Jinan, Shandong, China; ^3^ Key Laboratory of Endocrine Glucose & Lipids Metabolism and Brain Aging, Ministry of Education; Department of Neurology, Shandong Provincial Hospital Affiliated to Shandong First Medical University, Jinan, Shandong, China; ^4^ Medical Science and Technology Innovation Center, Shandong First Medical University & Shandong Academy of Medical Sciences, Jinan, Shandong, China; ^5^ Innovation Center for Neurological Disorders and Department of Neurology, Xuanwu Hospital, Capital Medical University, National Clinical Research Center for Geriatric Diseases, Beijing, Beijing, China; ^6^ Xuanwu Hospital Capital Medical University Jinan Branch, Jinan, Shandong, China; ^7^ Institute of Brain Science and Brain‐Inspired Research, Shandong First Medical University & Shandong Academy of Medical Sciences, Jinan, Shandong, China; ^8^ Aging Research Center, Department of Neurobiology, Care Sciences and Society, Karolinska Institute‐Stockholm University, Solna, Sweden

## Abstract

**Background:**

Despite high prevalence of multimorbidity in aging, associations with brain structures and covert vascular brain injury (VBI) remain unexplored.

**Method:**

This population‐based study included 1,264 dementia‐free participants (age≥60 years) in the MIND‐China MRI Substudy (2018–2020). We used hierarchical clustering to generate five multimorbidity clusters from 23 chronic diseases. We measured global structural brain markers (gray matter volume [GMV], white matter volume [WMV], cerebrospinal fluid [CSF] volume), regional AD‐related brain structural measures, and covert VBI markers (lacunes, cerebral microbleeds [CMBs], basal ganglia enlarged perivascular spaces [BG‐EPVS] and centrum semiovale enlarged perivascular spaces [CSO‐EPVS], and deep white matter hyperintensity [DWMH] and periventricular white matter hyperintensity [PWMH]). Data were analyzed using multiple linear regression models, ordinal logistic regression models, and partial correlation analyses.

**Result:**

Having multimorbidity was associated with β‐coefficient of ‐4.40 (95% CI, ‐8.02 to ‐0.79) for GMV, ‐4.54 (‐8.68 to ‐0.40) for WMV, 8.61 (2.01 to 15.20) for CSF volume, ‐0.20 (‐0.39 to ‐0.02) for precuneus volume, and ‐0.20 (‐0.39 to ‐0.02) for fusiform gyrus, 0.07 (0.04 to 0.11) for number of lacunes, and 0.03; 0.003 to 0.06) for number of CMBs, and increased likelihoods of BG‐EPVS (odds ratio=1.32; 1.06 to 1.64), PWMH Fazekas score (1.34; 1.08 to 1.66), DWMH Fazekas score (1.33; 1.07 to 1.66), and total cerebral small vascular disease (CSVD) burden (1.61; 1.30 to 1.98). The number of chronic diseases was associated with GMV (β=‐2.45; 95% CI, ‐3.90 to ‐0.99), WMV (‐2,73; ‐4.40 to ‐1.06), CSF volume (4.91; 2.25 to 7.57), precuneus volume (‐0.10; ‐0.17 to ‐0.02), fusiform gyrus (‐0.10; ‐0.15 to ‐0.05), middle temporal gyrus (‐0.12; ‐0.21 to ‐0.03), angular gyrus (‐0.07; ‐0.14 to 0.01), lacunes (0.03; 0.02 to 0.05), CMBs (0.02; 0.01 to 0.03), BG‐EPVS (odds ratio=1.13; 1.04 to 1.23), PWMH Fazekas score (1.21; 1.11 to 1.32), DWMH Fazekas score (1.12; 1.02 to 1.22) and total CSVD burden (1.25; 1.15 to 1.36). In addition, metabolic cluster, and cardiac‐musculoskeletal cluster were also significantly related to brain atrophy and VBI markers.

**Conclusion:**

Structural brain alterations of multimorbidity in older adults are characterized by global brain atrophy, atrophy in AD‐related brain regions, and covert VBI.

